# Characterization of the Polar Profile of *Bacon* and *Fuerte* Avocado Fruits by Hydrophilic Interaction Liquid Chromatography–Mass Spectrometry: Distribution of Non-structural Carbohydrates, Quinic Acid, and Chlorogenic Acid between Seed, Mesocarp, and Exocarp at Different Ripening Stages

**DOI:** 10.1021/acs.jafc.2c08855

**Published:** 2023-03-29

**Authors:** María Gemma Beiro-Valenzuela, Irene Serrano-García, Romina P. Monasterio, María Virginia Moreno-Tovar, Elena Hurtado-Fernández, José Jorge González-Fernández, José Ignacio Hormaza, Romina Pedreschi, Lucía Olmo-García, Alegría Carrasco-Pancorbo

**Affiliations:** † Department of Analytical Chemistry, Faculty of Sciences, University of Granada, Ave. Fuentenueva s/n, Granada 18071, Spain; ‡ Department of Biological and Health Sciences, Faculty of Health Sciences, Universidad Loyola Andalucía, Avda. de las Universidades s/n, Dos Hermanas, Sevilla 41704, Spain; § Facultad de Ciencias Agrarias, Instituto de Biología Agrícola de Mendoza (IBAM), UNCuyo—CONICET, Chacras de Coria, Mendoza 5505, Argentina; ∥ Institute for Mediterranean and Subtropical Horticulture (IHSM La Mayora-UMA-CSIC), Algarrobo-Costa, Málaga 29750, Spain; ⊥ Facultad de Ciencias Agronómicas y de los Alimentos, Escuela de Agronomía, Pontificia Universidad Católica de Valparaíso, Calle San Francisco S/N, La Palma, Quillota 2260000, Chile; # Millennium Institute Center for Genome Regulation (CRG), Santiago 8331150, Chile

**Keywords:** avocado tissues, C6 sugars, C7 sugars, fruit ripening, hydrophilic interaction chromatography−mass spectrometry, metabolite distribution

## Abstract

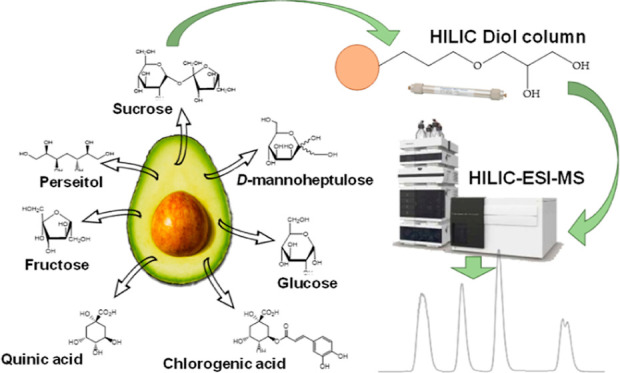

Avocado fruit growth and development, unlike that of other fruits, is characterized by the accumulation of oil and C7 sugars (in most fruits, the carbohydrates that prevail are C6). There are five essential carbohydrates which constitute 98% of the total content of soluble sugars in this fruit; these are fructose, glucose, sucrose, d-mannoheptulose, and perseitol, which together with quinic acid and chlorogenic acid have been the analytes under study in this work. After applying an efficient extraction procedure, a novel methodology based on hydrophilic interaction liquid chromatography coupled to mass spectrometry was applied to determine the levels of these seven substances in tissues—exocarp, seed, and mesocarp—from avocado fruits of two different varieties scarcely studied, *Bacon* and *Fuerte*, at three different ripening stages. Quantitative characterization of the selected tissues was performed, and the inter-tissue distribution of metabolites was described. For both varieties, d-mannoheptulose was the major component in the mesocarp and exocarp, whereas perseitol was predominant in the seed, followed by sucrose and d-mannoheptulose. Sucrose was found to be more abundant in seed tissues, with much lower concentrations in avocado mesocarp and exocarp. Quinic acid showed a predominance in the exocarp, and chlorogenic acid was exclusively determined in exocarp samples.

## 1 Introduction

Avocado (*Persea americana* Mill.) is a climacteric fruit that is native to Mexico and Central America. The first evidence of its consumption dates back to 8000–7000 C.E. in the Coxcatlan cave, Tehuacan Valley (state of Puebla) in Mexico.^1^ Three main ecological races of avocado are recognized: Mexican, Guatemalan, and West Indian. The first two subspecies are typical to tropical highlands, where colder conditions predominate, whereas the West Indian subspecies is originated from tropical lowlands, where the conditions are warmer. There is a wide variety of hybrids among the different subspecies producing fruits that present different physical and sensory properties, including different fruit maturity rates, oil percentages, etc.^2^

Avocado is a fruit with a high metabolic rate that completes its ripening in approximately 7 days at 25 °C after harvest,^3^ although this period is highly variable, depending on the variety and the maturity stage of the fruit when it is harvested. Unlike other fruits, avocado does not ripen on the tree; what happens is that several days after harvesting, the mesocarp softens and improves its organoleptic properties, becoming a palatable product for human consumption.^4,5^ During this process, some typical changes are observed, including external color modification (depending on the variety), texture alteration, and changes in the content of sugars, organics acids, and volatile compounds involved in nutritional quality, flavor, and aroma.^6^ In the early stages of avocado fruit growth and development, more than 40% of the mesocarp weight is made up of sugars.^7^ A characteristic of this fruit is the large amount of the less common heptose (C7) sugars (mannoheptulose and its polyol form, perseitol), which act as respiratory substrates, instead of hexose (C6) sugars (fructose, glucose, sucrose, etc.), as is typical in most fruits.^8−10^ Despite the importance of these carbohydrates, there are still many aspects to be unraveled about their synthesis, metabolism, transport, and physiological roles. Interesting studies postulate that perseitol acts as a storage sugar (energy source) and d-mannoheptulose as a transport sugar and sometimes as an energy supplier in the subsequent elicitation of other primary compounds.^8,10−13^ The implication of d-mannoheptulose as a potent inhibitor of hexokinase has also been suggested, preventing the entry of glucose into glycolysis, which hinders fruit ripening.^14^ It has also been reported that these C7 sugars act as antioxidants in the avocado mesocarp, and, therefore, their initial content at harvest correlates with fruit storability conferring not only carbohydrates to maintain respiration but also protective agents against stress.^15^

The determination of sugars represents an analytical challenge due to their polar nature and the absence of chromophore groups;^16^ similar is the case of amino acids and organic acids. For decades, they have been determined using gas chromatography coupled to mass spectrometry (GC–MS) or high-performance liquid chromatography (HPLC) coupled to different detection systems.^17^ GC–MS allows the simultaneous quantification of a large number of polar metabolites, with relatively low instrumental cost and the possibility of using libraries for compound identification. However, in many cases, a derivatization process is required to provide the compounds with sufficient volatility and thermostability.^17,18^ As far as HPLC is concerned, the separation of sugars has usually been performed with monosaccharide or NH_2_ columns (among others); sometimes, pre- or post-column derivatization reactions are resorted to, either to improve chromatographic separation or to ensure detectability.^19^ Refractive index detector (RID) and evaporative light scattering detector (ELSD) are very suitable detectors for the determination of sugars, although various LC–MS methods have been developed over the last 10 years with the aim to quantify one or more classes of polar compounds in different matrices.^17^ Recently, research using hydrophilic interaction liquid chromatography (HILIC) separations has increased substantially, and a number of stationary phases have been developed for this kind of chromatography. This is mainly due to the growing need to analyze hydrophilic metabolites in a wide variety of scientific fields and to the fact that HILIC provides enhanced retention and separation for polar compounds and is highly compatible with MS.^17,20^ HILIC consists of a polar chromatographic surface (bare silica or silica gels modified with many polar functional groups) with a mobile phase that includes a water-miscible polar organic solvent (such as acetonitrile) mixed with water, starting with a high percentage of organic solvent and ending with a high proportion of aqueous phase.^17,20,21^ Interesting applications to determine polar compounds in diverse matrices can be found in the literature.^16,18,22^

Focusing on the determination of carbohydrates and other polar compounds in avocado, Table S1—Supporting Information includes a good number of interesting works that provide an overview of the most considered avocado tissues and varieties, metabolites, and methodologies. As can be seen, there are several meritorious works dealing with the quantification of sugars and organic acids; however, some of them have considered the determination of very few compounds or required the use of two different analytical techniques or two chromatographic columns or entailed laborious sample preparation protocols (in some cases, defatting steps and/or clean-up procedures with solid-phase extraction). No work, so far, has applied a HILIC–MS methodology for the determination of carbohydrates, chlorogenic acid, and quinic acid in avocado tissues. In addition, most of the previous works have been performed mainly in *Hass*, the preponderant variety in the avocado market worldwide and, consequently, information is lacking for most other avocado varieties. *Bacon* and *Fuerte* are green skin varieties used in many countries as pollinizer for *Hass*.

This work was approached with multiple objectives: (i) to evaluate the potential of HILIC–MS to determine polar compounds in avocado tissues; (ii) to characterize samples of mesocarp, exocarp, and seed of *Bacon* and *Fuerte* varieties, determining the quantitative levels of C6 (fructose, glucose, and sucrose) and C7 (d-mannoheptulose and perseitol) sugars; and (iii) to describe the distribution of carbohydrates together with quinic acid and chlorogenic acid in avocado fruit tissues at three different ripening stages. It is evident that knowing in detail the composition of the different avocado tissues, the distribution of polar metabolites between tissues of the same fruit and how the ripening process affects them is of undeniable importance and contributes to improve the knowledge of significant physiological aspects of this tropical fruit. Therefore, this report presents an attractive analytical solution—avoiding the drawbacks of other methods—for the simultaneous determination of compounds of interest in avocado and contributes to describe avocado compositionally and to learn more about the primary post-harvest metabolic processes.

## 2 Materials and Methods

### 2.1 Chemicals and Reagents

For the preparation of the mobile phases, doubly deionized water with a conductivity of 18.2 MΩ obtained through a Milli-Q system (Millipore, Bedford, USA) was used. Acetonitrile LC–MS grade (ACN) was purchased from Lab-Scan (Dublin, Ireland), and ammonium acetate was provided by Sigma-Aldrich (St Louis, USA). Mobile phases were filtered using a 0.45 μm Nylaflo nylon membrane acquired from Pall Corporation (Michigan, USA). Extraction of the analytes of interest was carried out by using aqueous mixtures of ethanol (EtOH) from Prolabo (Paris, France). Standards of d-mannoheptulose (CAS number 3615-44-9), fructose (57-48-7), glucose (50-99-7), sucrose (57-50-1), quinic acid (77-05-2), and chlorogenic acid (327-97-9) acid were supplied by Sigma-Aldrich (St. Louis, MO, USA). Perseitol (527-06-0) was acquired from Carbosynth (Berkshire, United Kingdom). Stock solutions were prepared with specific concentrations of each metabolite in order to cover the appropriate quantitative ranges for each kind of avocado tissue (more details in Section 2.5). All prepared solutions and extracts were filtered using a nylon syringe filter (0.22 μm) Clarinet from Bonna-Agela Technologies (Wilmington, DE, USA) and stored in amber HPLC vials at −20 °C before injection.

### 2.2 Samples

The samples were obtained from a private avocado commercial orchard located in Vélez de Benaudalla, a municipality in the province of Granada, Spain. The coordinates of the orchard are latitude: 36° 49′ 55″, North longitude: 3° 30′ 58″ West, with an altitude above sea level of 171 meters.

The determination of the aforementioned analytes was carried out in three tissues—seed, exocarp, and mesocarp—of *Bacon* and *Fuerte* avocados. For both varieties, three different ripening stages were defined: freshly picked fruits (firmness range >50 N), fruits in an intermediate stage of ripening (50–15 N), and ready-to-eat fruits (edible ripeness; firmness <5 N). Fruit ripening took place under identical ambient conditions (20 ± 2 °C). Eighteen fruits were taken (three of each variety at each stage of ripening) which led, considering the three tissues, to a total of 54 samples. The samples were peeled, chopped, frozen, freeze-dried, crushed, homogenized, and stored at −20 °C. Each avocado fruit was considered independently, i.e., each avocado was processed and sampled and led to three samples (exocarp, mesocarp, and stone) to properly study the distribution of analytes among tissues of a single fruit. Considering that each avocado fruit is a unique specimen, it seemed appropriate to treat them as independent, since one of the main goals of this work was to explore the distribution of polar metabolites among tissues of the same fruit.

*Bacon* fruits were harvested in late October 2020 and *Fuerte* fruits at the end of November 2020. Percentage of dry matter (DM) was evaluated according to the AOAC 920.151 method^23^ as soon as the fruits were detached from the tree by taking, at least, 10–15 fruits and calculating the mean DM value. DM values for *Bacon* and *Fuerte* samples were between 23 and 25% (SD of DM measurements were close to 1 approx.). The DM values found can be considered normal for those avocado varieties in Spain at this time of the harvest season. According to EU regulations, avocados can already be harvested with 21% DM.

### 2.3 Extraction Procedure

Metabolites were extracted from 0.20 g of lyophilized samples and were mixed with 6 mL of EtOH/H_2_O, 60:40 (v/v). The mixture was shaken in vortex for 3 min; after that, the tubes were introduced into an ultrasound bath for 30 min, followed by centrifugation at 9000 rpm for 5 min. Once the solid phase and the supernatant were properly separated, the latter was transferred to a flask. The solid residue was extracted a second time, applying the same procedure (a second extraction cycle). Both supernatants were mixed and shaken in vortex for 1 min. Finally, approx. 1 mL of the solution was filtered and transferred to an HPLC vial. Two independent analytical replicates were prepared for each sample.

### 2.4 HILIC–MS Analyses

As the instrumental platform, a 1260 Infinity HPLC system (Agilent Technologies, Waldbronn, Germany) and an Esquire 2000 Ion Trap (IT) MS (Bruker Daltonics, Bremen, Germany) coupled by means of an electrospray ionization (ESI) source were used. Chromatographic separation of the different analytes of interest was performed using a Fortis HILIC-Diol column (Fortis Technologies, Cheshire, UK), whose dimensions were 2.1 × 150 mm and 1.7 μm particle size. The column operated at 25 °C, and the injection volume was set at 2 μL. The chromatographic flow was fixed at 0.3 mL/min. Mobile phases were prepared with water and ACN at different proportions: H_2_O/ACN (95:5, v/v) for phase A and H_2_O/ACN (5:95, v/v) for phase B. Ammonium acetate buffer was added to both phases to have the same final concentration in both bottles (10 mM). The applied elution conditions were: 0 min, 2% A and 98% B; 5 min, 2% A and 98% B (5 min at isocratic conditions); 20 min, 35% A and 65% B; at 21.5 min, the system returned to initial conditions. Each analysis lasted approximately 30 min, taking into account column reequilibration. The MS was operated in a negative mode, and data were acquired in a full scan mode for a mass range from 50 to 1000 *m*/*z*. In order to achieve stable ionization, the nebulizer gas (nitrogen) was set at 30 psi, dry gas (nitrogen) flow rate at 9 L/min, and temperature at 300 °C. The optimum capillary voltage was established at +3200 V, and the end-plate offset at −500 V.

The software used to control the LC and MS systems were Agilent ChemStation (Agilent Technologies) and Esquire Control (Bruker Daltonics), respectively. In addition, data processing, management, and representations were performed by using DataAnalysis 4.0 software (Bruker Daltonics, Bremen, Germany) and Microsoft Excel v 2204.

### 2.5 Establishing the Analytical Parameters of the Method

Pure standard solutions as well as avocado tissues extracts were used for the validation of the method. Linearity, precision, and recovery of the extraction protocol were evaluated.

Solutions of the seven pure compounds (perseitol, d-mannoheptulose, fructose, glucose, sucrose, and quinic acid, and chlorogenic acid) were prepared in EtOH/H_2_O, 60:40 (v/v) at ten different concentration levels (over the range from the quantification limit to the maximum considered concentration level for each substance) to establish external calibration curves. The concentration of the stock solutions ranged, approximately, from 0.05 to 250 mg/L for fructose and glucose, from 0.05 to 500 mg/L for d-mannoheptulose, chlorogenic, sucrose, and quinic acid, and 0.1–1350 mg/L for perseitol. Two specific working quantitative ranges or linear dynamic ranges were established for each metabolite, except for chlorogenic acid.

Detection and quantification limits (LOD and LOQ) of each analyte were calculated using the signal/noise ratio (*S*/*N*) obtained at the lowest concentration level injected (which was different for each compound), estimating the concentration that generated a *S*/*N* equal to 3 and 10, respectively.

Repeatability (intra-day and inter-day) was considered to assess the precision of the method; both values were expressed as coefficient of variation (% CV). The intra-day repeatability was obtained from five injections of a standard mix (containing the seven selected metabolites) and a quality control (QC) sample (prepared by mixing an aliquot of extracts from the three tissues) carried out within the same sequence, while inter-day repeatability was obtained from 12 injections performed in different sequences.

The recovery (expressed as percentage) was estimated by subjecting samples that had already been extracted, as described in Section 2.3, to a third extraction cycle and evaluating whether detectable amounts of the metabolites of interest were found; in other words, by checking whether any remaining amounts of the target substances were left in the pellet. Method trueness was evaluated by analyzing samples extracted before and after spiking known concentrations of pure standards and measuring the discrepancy between the obtained results. Moreover, possible matrix effects were assessed by comparing the slope of a standard addition curve (in a mix of exocarp, mesocarp, and seed extracts) and the external calibration curve and calculating a matrix effect coefficient as follows^24^
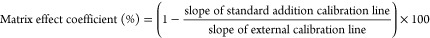


In general, it has been established that the matrix effect can be considered as negligible if the matrix effect coefficient is found within a range of ±20%.

### 2.6 Statistical Analysis

The data were statistically analyzed using Statgraphics 19 (Statgraphics Technologies, Inc., The Plains, VA, USA). A one-way analysis of variance (ANOVA) was performed to compare the results of each analyte for the three avocados of the same ripening stage and another one to compare fruits belonging to different ripening stages. The significance of the differences at 5% (*p* < 0.05) level between mean values was determined using the Tukey’s test.

## 3 Results and Discussion

### 3.1 Selection of Experimental Conditions and Qualitative Profile of Avocado by HILIC–MS

As concerns the extraction procedure, several conditions were evaluated, making changes in the nature of the extractant agents, number of extraction cycles, sample amount, and extractant volume. In addition, three different extraction systems (ultrasound assisted, vortex shaking, and heating bath) were tested. The objective was to select the most suitable protocol from those considered, which would be valid for all chosen metabolites and would result in good reproducibility. All evaluated conditions are summarized in Table S2—Supporting Information and the selected protocol is described in Section 2.3. On the other hand, with the aim of achieving good chromatographic resolution in a reasonable analysis time, different chromatographic conditions were also tested. Table S3—Supporting Information shows the parameters considered for the tests, including the elution gradient, flow rate, injection volume, and column type. The conditions that provided the most favorable separation are described in Section 2.4.

As a first step, the qualitative examination of the chromatographic profiles obtained was carried out. Table 1 includes the retention time of each compound, the signal (or signals) generated in MS as well as their assigned identity. For all compounds, a pure commercial standard was available, so the identification was done by comparing retention times, MS response, and also by spiking the extracts of the different tissues. Previously published reports were also considered.^5,25,26^ All analytes ionized, giving a prominent signal corresponding to their pseudo-molecular ion, except chlorogenic acid, which also showed a distinctive in-source fragment with *m*/*z* 191 ([M – H-162]^−^). As it will be described in the following sections, all selected metabolites were determined in the three fruit tissues of both varieties, except for chlorogenic acid, which was only quantified in the avocado exocarp.

**Table 1:** Metabolites Detected in Avocado Extracts, Together with Their Retention Time, MS signal/s, Molecular Formula, and Assigned Identity (Corroborated with Pure Standards)^a^

Rt (min)	detected *m*/*z*^b^	molecular formula	assignment
6.6	179 [M – H]^−^	C_6_H_12_O_6_	fructose
8.7	179 [M – H]^−^	C_6_H_12_O_6_	glucose
11.2	209 [M – H]^−^	C_7_H_14_O_7_	d-mannoheptulose
13.5	353 [M – H]^−^, 191 [M – H]^−^	C_16_H_18_O_9_	chlorogenic acid
16.7	211 [M – H]^−^	C_7_H_16_O_7_	perseitol
18.3	341 [M – H]^−^	C_12_H_22_O_11_	sucrose
18.9	191 [M – H]^−^	C_7_H_12_O_6_	quinic acid

a Abbreviation: Rt (retention time).

b When more than one *m*/*z* signal is included, they are listed in the order of decreasing intensity.

The elution order of the selected analytes was as follows: fructose, glucose, d-mannoheptulose, chlorogenic acid, perseitol, sucrose, and quinic acid. Owing to the use of a HILIC stationary phase and the selected elution gradient, the compounds that eluted in the first minutes were those with a more moderate polarity, and those that eluted in the last part of the analysis were those with the highest polarity.

Initially, the method was intended to exclusively determine C6 and C7 sugars; however, when exocarp, mesocarp, and seed extracts were analyzed, we observed that there were other interesting metabolites that could be determined along with sugars. This led to the inclusion of quinic acid and chlorogenic acid as well. The relevance of these two substances in plants is beyond dispute. Quinic acid, among other functions, contributes to the sugar/acid balance and health-giving properties of the fruits. Moreover, the content of organic acids in fruits is closely associated with the activities of the related metabolic enzyme.^27^ The term “chlorogenic acids” encompasses a large group of naturally occurring compounds of which the majority are synthesized in plants by esterification of a C6–C3 *trans*-hydroxycinnamic acid with 1l-(−)-quinic acid.^28^ Many of these compounds, like other polyphenols, are associated with important health benefits and well-known as nutritional antioxidants in plant foods. It is not easy to describe unambiguously the structures of acyl-quinic acids that may appear almost identical when drawn in 2D or projected in 3D.^29−31^ In this paper, we focus on the determination of a relevant compound of this category, which is assigned the trivial name of chlorogenic acid (CAS number 327-97-9).^28^Figure S1—Supporting Information shows the extracted ion chromatograms (EICs) of the target analytes in (A) a standard mix and in (B) an example of an exocarp avocado extract. It seems pertinent to indicate that some other compounds were detected in our analytical window (among them, some isomers of *m*/*z* 353 in seed extracts); however, as we were unable to assign a tentative identity, these analytes were not further considered.

### 3.2 Analytical Parameters of the Method

The applied methodology was evaluated considering the analytical parameters previously described. The numerical results appear in Table 2, where the equations of the calibration curve for each linear concentration range, LOD and LOQ, intra- and inter-day repeatability values (% CV), recovery of the extraction protocol, trueness, and matrix effect coefficient for each compound are included.

**Table 2:** Analytical Parameters of the HILIC–MS Method Used in the Current Study^a^

						repeatability intra-day (% CV)^b^		repeatability inter-day (% CV)^c^				
compound	calibration curve	*r*^2^	lineal range (mg/L)	LOD (mg/L)	LOQ (mg/L)	standard mix	QC sample	standard mix	QC sample	recovery (% *R*)^d^	trueness (% *R*)^e^	matrix effect coefficient (%)^f^
fructose	*y* = 1110.3*x* + 1317	0.9948,	LOQ-67	0.09	0.29	3.6	4.0	9.6	10.5	100.0	99.9	14.40
	*y* = 838.7*x* + 27939	0.9948	67–267									5.18
glucose	*y* = 1324.1*x* + 4422.6	0.9945	LOQ-67	0.06	0.22	2.2	3.5	6.1	6.7	100.0	92.3	8.97
	*y* = 793.82*x* + 42613	0.9891	67–267									–3.96
d-mannoheptulose	*y* = 977.04*x* + 582	0.9979,	LOQ-125	0.10	0.34	6.1	5.1	10.5	9.8	100.0	95.9	12.85
	*y* = 720.03*x* + 27488	0.9986	125–500									–10.60
chlorogenic acid	*y* = 9748.5*x* – 494078	0.9950	32–500	0.02	0.08	4.4	4.8	10.9	12.1	100.0	99.2	1.78
perseitol	*y* = 2906.8*x* + 10305	0.9943	LOQ-167	0.03	0.10	1.8	2.3	5.8	5.3	99.9	101.1	–2.12
	*y* = 1705.5*x* + 264917	0.9987	167–1334									–8.53
sucrose	*y* = 992.82*x* + 2171.1	0.9921	LOQ-62.5	0.10	0.32	1.2	1.9	9.1	10.0	99.9	102.2	12.31
	*y* = 414.85*x* + 43732	0.9939	62.5–500									1.14
quinic acid	*y* = 4195.3*x* – 3368.5	0.9935	LOQ-34.4	0.01	0.04	11.4	10.3	7.9	5.0	97.7	98.4	11.4
	*y* = 3636.9*x* + 129413	0.9921	34.4–550									–2.58

a Abbreviations used: LOD (limit of detection); LOQ (limit of quantification); CV (coefficient of variation).

b Coefficient of variation (%) corresponding to injections (*n* = 5) of a standard mix (of intermediate concentration) and a QC sample (mix of exocarp, seed, and mesocarp extracts) carried out in the same sequence.

c Coefficient of variation (%) corresponding to injections (*n* = 12) of a standard mix (of intermediate concentration) and a QC sample (mix of exocarp, seed, and mesocarp extracts) performed in sequences carried out on 5 consecutive days.

d Recovery (%) was measured by applying a third extraction cycle to a QC sample (mix of exocarp, seed, and mesocarp extracts). When 100% is indicated, it means that a quantifiable signal of the analyte was not observed, so it was assumed that the substance had been completely extracted with the previous two extraction cycles.

e Trueness (%) in the QC sample extracted before and after the addition of known concentrations of standards. The values included in this table are those obtained for an intermediate concentration level of all those tested.

f Matrix effect coefficient (%) calculated by comparing slopes of two calibration curves (external and standard addition on the QC sample extract).

The LODs obtained ranged between 0.01 and 0.10 mg/L and the LOQs between 0.04 and 0.34 mg/L for quinic acid and d-mannoheptulose, respectively. The intra-day repeatability, in all cases, presented values lower than 11.4%, whereas inter-day repeatability was consistently below 12.1%. These results can be considered quite adequate, as it should be noted that HILIC methodologies are generally somewhat less robust than those employing reversed-phase LC. The extraction protocol implemented was also satisfactory, since it led to recovery values ranging from 97.7 to 100%. The trueness of the method was found between 92.3 and 102.2%, and the matrix effect coefficients varied from −10.60 to 14.40 for d-mannoheptulose and fructose, respectively, which means that enhancing or suppressing effects were negligible.

### 3.3 Characterization of the Quantitative Polar Profile of Three Fruit Tissues from *Bacon* and *Fuerte* Avocado Varieties

After verifying that the developed method for the determination of C6 and C7 carbohydrates, as well as quinic acid and chlorogenic acid in avocado tissues, showed acceptable analytical parameters, we proceeded to quantify these analytes in the 108 extracts (three biological replicates per ripening stage, three tissue samples per fruit, three ripeness stages, two varieties, and two technical replicates). The quantitative results (expressed in mg of analyte/g of tissue) are shown with their corresponding standard deviation in Table 3 (Table 3A includes results for *Bacon* and Table 3B for *Fuerte*).

**Table 3A:** Content of Sugars, Chlorogenic Acid, and Quinic Acid (Expressed as mg Metabolite/g Tissue; Mean ± SD) in Exocarp, Seed, and Mesocarp of *Bacon* Avocado Fruits^a^

(A) *Bacon*									
	unripe			intermediate ripening			ready-to-eat		
**Exocarp**									
chlorogenic acid	3.32 ± 0.01^ab1^	3.22 ± 0.03^a1^	3.41 ± 0.01^b1^	3.8 ± 0.2^a2^	3.93 ± 0.05^a2^	3.6 ± 0.1^a2^	4.15 ± 0.04^a3^	4.21 ± 0.02^a3^	4.20 ± 0.02^a3^
fructose	0.84 ± 0.09^a1^	1.1 ± 0.2^a1^	1.1 ± 0.1^a1^	3.3 ± 0.6^a2^	2.3 ± 0.4^a2^	1.9 ± 0.5^a2^	1.14 ± 0.05^a1^	1.7 ± 0.2^a1^	1.4 ± 0.1^a1^
glucose	1.37 ± 0.06^a1^	1.0 ± 0.2^a1^	1.3 ± 0.1^a1^	2.6 ± 0.3^a3^	1.8 ± 0.1^a3^	2.2 ± 0.2^a3^	1.46 ± 0.06^b2^	2.03 ± 0.01^a2^	1.70 ± 0.05^b2^
d-mannoheptulose	32 ± 3^b1^	6 ± 1^a1^	21 ± 2^b1^	30 ± 5^a1^	20 ± 4^a1^	24 ± 4^a1^	22 ± 4^a1^	31 ± 3^a1^	28 ± 3^a1^
perseitol	1.90 ± 0.04^a2^	4.2 ± 0.5^b2^	2.8 ± 0.3^ab2^	0.26 ± 0.09^a1^	0.50 ± 0.06^a1^	0.37 ± 0.07^a1^	0.19 ± 0.03^a1^	0.120 ± 0.004^a1^	0.15 ± 0.01^a1^
quinic acid	14.3 ± 0.5^a1^	13 ± 2^a1^	14 ± 1^a1^	19 ± 4^a1^	17 ± 3^a1^	14 ± 3^a1^	18 ± 4^a1^	17 ± 3^a1^	17 ± 1^a1^
sucrose	0.10 ± 0.02^a1^	0.9 ± 0.2^b1^	0.1 ± 0.1^a1^	1.6 ± 0.3^a2^	1.6 ± 0.1^a2^	1.5 ± 0.1^a2^	0.9 ± 0.1^a2^	1.9 ± 0.2^b2^	1.4 ± 0.2^ab2^
**Seed**									
fructose	2.54 ± 0.06^a1^	1.6 ± 0.3^a1^	2.0 ± 0.2^a1^	3.01 ± 0.04^a2^	4.5 ± 0.4^b2^	3.4 ± 0.2^ab2^	4.00 ± 0.06^a2^	3.9 ± 0.2^a2^	3.8 ± 0.1^a2^
glucose	1.47 ± 0.04^a12^	3.7 ± 0.7^b12^	1.5 ± 0.4^a12^	1.2 ± 0.2^a1^	0.66 ± 0.1^a1^	1.4 ± 0.1^a1^	7.6 ± 0.5^c2^	1.29 ± 0.2^a2^	3.8 ± 0.5^b2^
d-mannoheptulose	4.8 ± 0.5^b1^	1.9 ± 0.4^a1^	3.0 ± 0.4^ab1^	11 ± 2^a2^	15 ± 4^a2^	11 ± 3^a2^	13 ± 2^a2^	11.0 ± 0.2^a2^	11.2 ± 0.6^a2^
perseitol	79 ± 1^b1^	51 ± 5^a1^	52 ± 3^a1^	79 ± 14^a2^	82 ± 3^a2^	70 ± 8^a2^	78 ± 1^a2^	75 ± 2^a2^	78 ± 1^a2^
quinic acid	6.5 ± 0.2^b1^	1.9 ± 0.2^a1^	2.1 ± 0.2^a1^	10.4 ± 0.7^b1^	5 ± 1^a1^	10.8 ± 0.8^b1^	14 ± 2b1	3.80 ± 0.02^a1^	6 ± 1^a1^
sucrose	8 ± 1^a1^	20 ± 3^b1^	16 ± 2^ab1^	15 ± 2^a1^	16.9 ± 0.8^a1^	15 ± 1^a1^	13 ± 1^a1^	21.8 ± 0.3^b1^	19.5 ± 0.9^b1^
**Mesocarp**									
fructose	1.79 ± 0.04^a1^	2.8 ± 0.4^a1^	2.4 ± 0.2^a1^	4.7 ± 0.2^a2^	5.6 ± 0.3^a2^	4.9 ± 0.3^a2^	7.0 ± 0.8^a3^	7±1^a3^	5.6 ± 0.5^a3^
glucose	1.72 ± 0.05^a1^	3.54 ± 0.05^c1^	2.68 ± 0.05^b1^	7 ± 1^a2^	6 ± 1^a2^	6 ± 1^a2^	7.9 ± 0.5^a2^	6 ± 1^a2^	7.5 ± 0.7^a2^
d-mannoheptulose	63 ± 8^a1^	35 ± 5^a1^	43 ± 7^a1^	49 ± 9^a1^	49 ± 13^a1^	49 ± 7^a1^	57 ± 1^a1^	60 ± 6^a1^	54.6 ± 0.6^a1^
perseitol	3.7 ± 0.2^a2^	17 ± 2^b2^	12 ± 1^b2^	0.39 ± 0.02^a1^	1.0 ± 0.2^b1^	0.8 ± 0.1^ab1^	0.002 ± 0.001^a1^	0.31 ± 0.04^c1^	0.16 ± 0.02^b1^
quinic acid	5.7 ± 0.8^a1^	6 ± 1^a1^	5 ± 1^a1^	10 ± 1^a2^	9 ± 1^a2^	9 ± 1^a2^	9.2 ± 0.2^a2^	11.2 ± 0.6^a2^	10.2 ± 0.4^a2^
sucrose	0.46 ± 0.08^b1^	0.14 ± 0.04^ab1^	0.12 ± 0.06^a1^	0.56 ± 0.09^a2^	1.2 ± 0.1^a2^	0.7 ± 0.1^a2^	2.1 ± 0.2^a3^	2.18 ± 0.09^a3^	2.27 ± 0.02^a3^

a Different letters at the same line show statistical differences (*p* ≤ 0.05) among avocado fruits with the same ripening stage; different numbers at the same line show statistical differences (*p* ≤ 0.05) when comparing the same analyte at a distinct stage of ripening fruit.

**Table 3B:** Content of Sugars, Chlorogenic Acid, and Quinic Acid (Expressed as mg Metabolite/g Tissue; Mean ± SD) in Exocarp, Seed, and Mesocarp of *Fuerte* Avocado Fruits^a^

(B) *Fuerte*									
	unripe			intermediate ripening			ready-to-eat		
**Exocarp**									
chlorogenic acid	3.31 ± 0.03^a1^	4.9 ± 0.5^a1^	4.4 ± 0.2^a1^	4.0 ± 0.3^a1^	5.9 ± 0.4^b1^	4.2 ± 0.3^a1^	5.6 ± 0.2^a2^	6.0 ± 0.2^a2^	6.0 ± 0.5^a2^
fructose	1.4 ± 0.2^a3^	0.89 ± 0.09^a3^	1.3 ± 0.1^a3^	0.89 ± 0.06^a2^	0.67 ± 0.08^a2^	0.68 ± 0.02^a2^	0.30 ± 0.02^ab1^	0.34 ± 0.01^b1^	0.141 ± 0.001^a1^
glucose	1.1 ± 0.1^a2^	0.79 ± 0.02^a2^	0.82 ± 0.07^a2^	0.15 ± 0.01^a1,2^	0.97 ± 0.09^b1,2^	0.30 ± 0.04^a1,2^	0.42 ± 0.03^a1^	0.19 ± 0.01^a1^	0.12 ± 0.01^a1^
d-mannoheptulose	20 ± 2^a1^	19 ± 2^a1^	18 ± 2^a1^	10 ± 1^a1^	24 ± 4^b1^	22 ± 3^b1^	7.8 ± 0.4^a1^	18.4 ± 0.8^b1^	12.6 ± 0.4^ab1^
perseitol	6.6 ± 0.6^b2^	2.6 ± 0.1^a2^	6.6 ± 0.4^b2^	0.10 ± 0.01^a1^	1.6 ± 0.1^b1^	0.21 ± 0.08^a1^	0.0010 ± 0.0001^a1^	0.0012 ± 0.0001^a1^	0.053 ± 0.001^b1^
quinic acid	10.7 ± 0.02^a1^	19 ± 2^b1^	17.6 ± 0.9^b1^	11 ± 1^a1^	18 ± 1^a1^	12 ± 1^a1^	16 ± 1^a1^	12.7 ± 0.5^a1^	14.2 ± 0.1^a1^
sucrose	0.8 ± 0.3^a2^	0.36 ± 0.02^a2^	0.7 ± 0.2^a2^	0.1201 ± 0.0002^a1,2^	0.47 ± 0.02^c1,2^	0.301 ± 0.002^b12^	0.102 ± 0.001^a1^	0.35 ± 0.01^b1^	0.143 ± 0.007^a1^
**Seed**									
fructose	3.7 ± 0.2^b1^	2.98 ± 0.01^a1^	3.3 ± 0.1^ab1^	2.98 ± 0.01^b1^	0.45 ± 0.04^a1^	0.51 ± 0.02^a1^	3.6 ± 0.1^a1^	5.7 ± 0.2^c1^	3.7 ± 0.2^b1^
glucose	0.64 ± 0.07^a1,2^	9.0 ± 0.8^b1,2^	4.4 ± 0.4^c1,2^	3.9 ± 0.1^a1^	1.18 ± 0.01^a1^	2.81 ± 0.08^b1^	0.81 ± 0.04^a2^	21.1 ± 0.9^b2^	10±1^ab2^
d-mannoheptulose	12 ± 1^a2^	10 ± 1^a2^	11 ± 1^a2^	4.1 ± 0.5^a1^	4.2 ± 0.4^a1^	3.83 ± 0.08^a1^	8.6 ± 0.7^a2^	18.9 ± 0.8^a2^	16 ± 1^a2^
perseitol	62.0 ± 0.8^a1^	91 ± 2^c1^	76 ± 1^b1^	75 ± 5^a1,2^	71 ± 2^a1,2^	70 ± 2^a1,2^	70 ± 6^a2^	54 ± 2^a2^	55 ± 1^a2^
quinic acid	5.7 ± 0.5^a1^	5.6 ± 0.2^a1^	5.5 ± 0.2^a1^	5.4 ± 0.1^b1^	4.43 ± 0.02^a1^	4.13 ± 0.08^a1^	5.9 ± 0.5^ab1^	6.0 ± 0.2^a1^	8.5 ± 0.7^b1^
sucrose	12 ± 1^a1^	7.6 ± 0.3^b1^	8.9 ± 0.6^ab1^	7.8 ± 0.6^a1^	9.12 ± 0.9^a1^	8.3 ± 0.8^a1^	17 ± 2^a2^	27 ± 1^b2^	23 ± 1^ab2^
**Mesocarp**									
fructose	1.83 ± 0.08^c1^	0.20 ± 0.02^a1^	1.01 ± 0.02^b^	0.104 ± 0.004^a1^	0.90 ± 0.08^c1^	0.4 ± 0.1^b1^	0.6 ± 0.1^b1^	0.17 ± 0.01^a1^	0.061 ± 0.0001^a1^
glucose	1.6 ± 0.1^c1^	0.40 ± 0.03^a1^	1.21 ± 0.07^c1^	0.21 ± 0.02^a1^	1.8 ± 0.1^b1^	1.4 ± 0.2^b1^	0.80 ± 0.07^b1^	0.21 ± 0.01^a1^	0.12 ± 0.01^a1^
d-mannoheptulose	17 ± 2^a2^	17 ± 2^a2^	17 ± 2^a2^	5.3 ± 0.4^a1,2^	18.9 ± 1.2^c1,2^	10.1 ± 0.9^b1,2^	4.3 ± 0.3^a1^	8.0 ± 0.3^b1^	3.4 ± 0.3^a1^
perseitol	8.9 ± 0.3^b2^	3.6 ± 0.2^a2^	3.8 ± 0.2^a2^	0.30 ± 0.01^a2^	2.52 ± 0.02^c1^	1.51 ± 0.01^b1^	0.0004 ± 0.0001^a1^	0.0007 ± 0.0002^a1^	0.01 ± 0.002^b1^
quinic acid	7.1 ± 0.2^a1^	11.3 ± 0.2^b1^	11.1 ± 0.2^b1^	6.8 ± 0.1^a1^	12.9 ± 0.2^c1^	7.87 ± 0.09^b1^	12.8 ± 0.2^b1^	5.4 ± 0.2^a1^	5.4 ± 0.1^a1^
sucrose	1.2 ± 0.1^a2^	2.20 ± 0.03^b2^	1.61 ± 0.08^a2^	0.105 ± 0.003^a1^	0.08 ± 0.01^ab1^	0.090 ± 0.001^b1^	0.011 ± 0.001^a1^	0.31 ± 0.01^b1^	0.301 ± 0.003^b1^

a Different letters at the same line show statistical differences (*p* ≤ 0.05) among avocado fruits with the same ripening stage; different numbers at the same line show statistical differences (*p* ≤ 0.05) when comparing the same analyte at a distinct stage of ripening fruit.

After applying one-way ANOVA analyses described above, it was observed that the concentration values of some analytes showed significant differences between avocado fruits at the same ripening stage, which is understandable, considering that each avocado fruit is a different specimen (an independent biological replicate); in most cases, however, no such significant differences were observed between fruits at the same ripening stage. When the comparison (one-way ANOVA) was made between the different ripening stages (i.e., unripe, intermediate stage of ripeness and ready-to-eat fruits), there were not many cases in which significant differences were observed between the quantitative results of an analyte for the three ripening stages. Significant differences were observed, for example, for chlorogenic acid and glucose in the exocarp of both varieties, for fructose in the exocarp of *Fuerte*, and for sucrose and fructose in the mesocarp of *Bacon*. Perseitol also exhibited some significant differences over ripening for the different tissues of the two varieties. However, as previously stated, the purpose of this work was not to formally study the evolution of these compounds along ripening but rather to establish their quantitative levels and their distribution among the different tissues of the same fruit (considering fruits at diverse ripening levels). Nevertheless, some remarks will be made in this regard (metabolites evolution over ripening) in future paragraphs or sections of this work.

As discussed in the Introduction, existing studies on the characterization of avocado sugars focus on assessing how these compounds fluctuate during the season (sampling at different time intervals) and measuring them in physiologically mature but still unripe fruit. Relatively few papers have been published dealing with ripening (different storage conditions and ripening intervals) and on C6 and C7 sugars,^8,9,13,32−37^ but what is still largely unknown is how C7 and C6 sugars are allocated to the different plant tissues in which they are found. In addition, most studies have been performed in *Hass* and, to the best of our knowledge, there is no publication describing the characterization of the polar compound profiles in *Bacon* and *Fuerte* (even less by using HILIC–MS).

**Figure 1:**
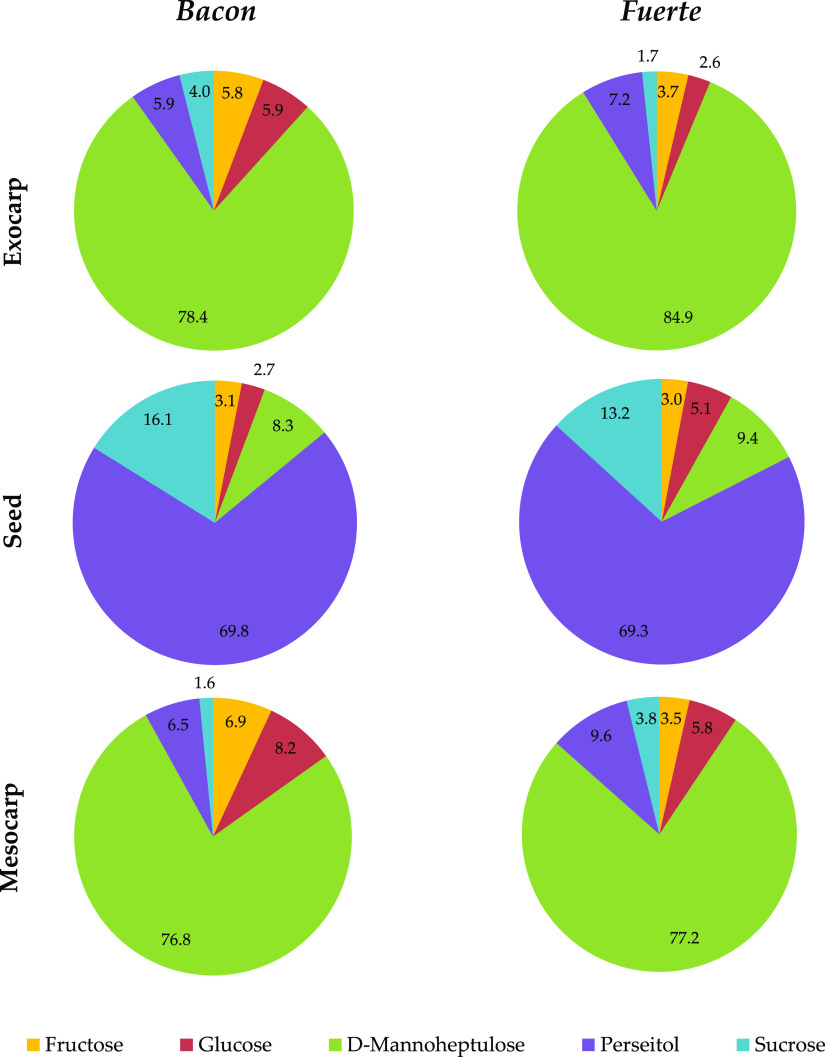
Pie charts showing the general composition (in percentage terms) of each matrix for *Bacon* and *Fuerte* varieties, averaging the results of the nine samples of each tissue.

Figure 1 illustrates in percentage terms (averaging the results of the nine samples of each tissue) the general composition of each matrix for fruits of *Bacon* and *Fuerte* varieties. The results show that d-mannoheptulose is the main component in the mesocarp (76.8% for *Bacon* and 77.2% for *Fuerte*) and exocarp (78.4% and 84.9% for *Bacon* and *Fuerte*, respectively), and perseitol is predominant in the seed (69.8% for *Bacon* and 69.3% for *Fuerte*), followed by sucrose and d-mannoheptulose. This statement can be made for both varieties and agrees with the published literature.^32,35,37,38^

If these percentages were calculated for each ripening stage, the representation would be quite similar for mesocarp and exocarp, but, in unripe avocados, there would be a considerably higher percentage of perseitol. In other words, the percentage of perseitol decreases in these matrices over the ripening process. For the seed, a decrease in the relative proportion of perseitol is also observed during the softening of the fruit (more marked in the *Fuerte* variety), accompanied by an increase of d-mannoheptulose in *Bacon* and of fructose and glucose in *Fuerte*.

When looking at the quantitative results of the metabolites under study included in Table 3A,B, it is possible to highlight that for the tissues for which previously published data are available, the results obtained for *Bacon* and *Fuerte* varieties are rather in agreement with those described for the *Hass* variety.^9,13,14,32−34,36,39,40^

We will begin by commenting on the results for exocarp, then for seed, and finally for mesocarp tissues. For *Bacon* exocarp, chlorogenic and quinic acids were found in concentrations within the range 3.32–4.21 and 13–19 mg/g, respectively. The values obtained for chlorogenic acid in exocarp tissues are on the order of those obtained for the *Hass* variety and substantially higher than those of *Creole* avocados.^41^ The values for C6 sugars in the exocarp of the *Bacon* variety ranged from 1.0 to 2.6 mg/g for glucose, from 0.84 to 3.3 mg/g for fructose, and from 0.10 to 1.9 mg/g for sucrose. As far as C7 carbohydrates are concerned, d-mannoheptulose was always found at concentration levels notably higher than those of perseitol (6–32 mg/g for d-mannoheptulose and 0.120–4.2 mg/g for perseitol). A rather similar situation was observed for the samples of the same tissue from *Fuerte* avocados. Possibly, the most marked differences between the two varieties were detected when comparing sucrose levels in the exocarp, sucrose concentrations being slightly lower in *Fuerte*.

In the seed, the ranges found for quinic acid were 1.9–14 mg/g in *Bacon* and 4.13–8.5 in *Fuerte*. Seed fructose levels were similar for both varieties, as were sucrose levels, with a slight overall increase in the concentrations of this analyte in both varieties throughout ripening (particularly in *Fuerte*). For the C7 metabolites, the situation was opposite to that observed in the exocarp, since the concentration of perseitol in the seed was higher than that of d-mannoheptulose, as previously described in the literature.^42^ The values obtained were 51–82 and 1.9–15 mg/g for perseitol and d-mannoheptulose, respectively, in *Bacon*, and 54–91 mg/g and 3.83–18.9 mg/g for the same analytes in *Fuerte*. A similar observation had been reported in *Hass* avocado seeds, indicating that perseitol was the most abundant carbohydrate, followed by sucrose, d-mannoheptulose, fructose, and glucose.^14^ Also, Tesfay et al., 2012, made statements in the same direction in a very interesting piece of work focused on the search for the function of carbohydrates in *Hass* avocados.^37^

The most notable differences between varieties were observed in mesocarp samples. *Bacon* showed higher C6 concentrations than those found in *Fuerte*, except for sucrose in unripe fruits. These sugars had a quite clear tendency to increase over ripening in *Bacon*; however, this tendency could not be corroborated for *Fuerte*. The evolution of C6 sugars over ripening needs to be studied further, since contradictory results are found in the literature depending on the variety and the experimental design of the study.^13,14,33,37^d-Mannoheptulose levels were consistently higher in samples of *Bacon* variety. Perseitol concentration decreased in both varieties during fruit softening. Quinic acid concentrations determined for *Bacon* samples fluctuated from 5 to 11.2 mg/g (increasing slightly during ripening) and from 5.4 to 12.9 mg/g for *Fuerte* fruits (no clear tendency could be established in this case).

In order to clarify the results discussed in this section, Figure S2—Supporting Information describes the average composition of each tissue for *Bacon* and *Fuerte* varieties at each ripening level by using radar charts; for better adaptation of the scales and to make visual comparison feasible, logarithmic axes have been used. The radar chart or spider chart offers the opportunity to display multivariate data in the form of a two-dimensional plot (on an axis starting at the center of the graph) of several quantitative variables.

### 3.4 Distribution of the Seven Determined Metabolites among Different Tissues of the Avocado Fruit over Ripening

**Figure 2:**
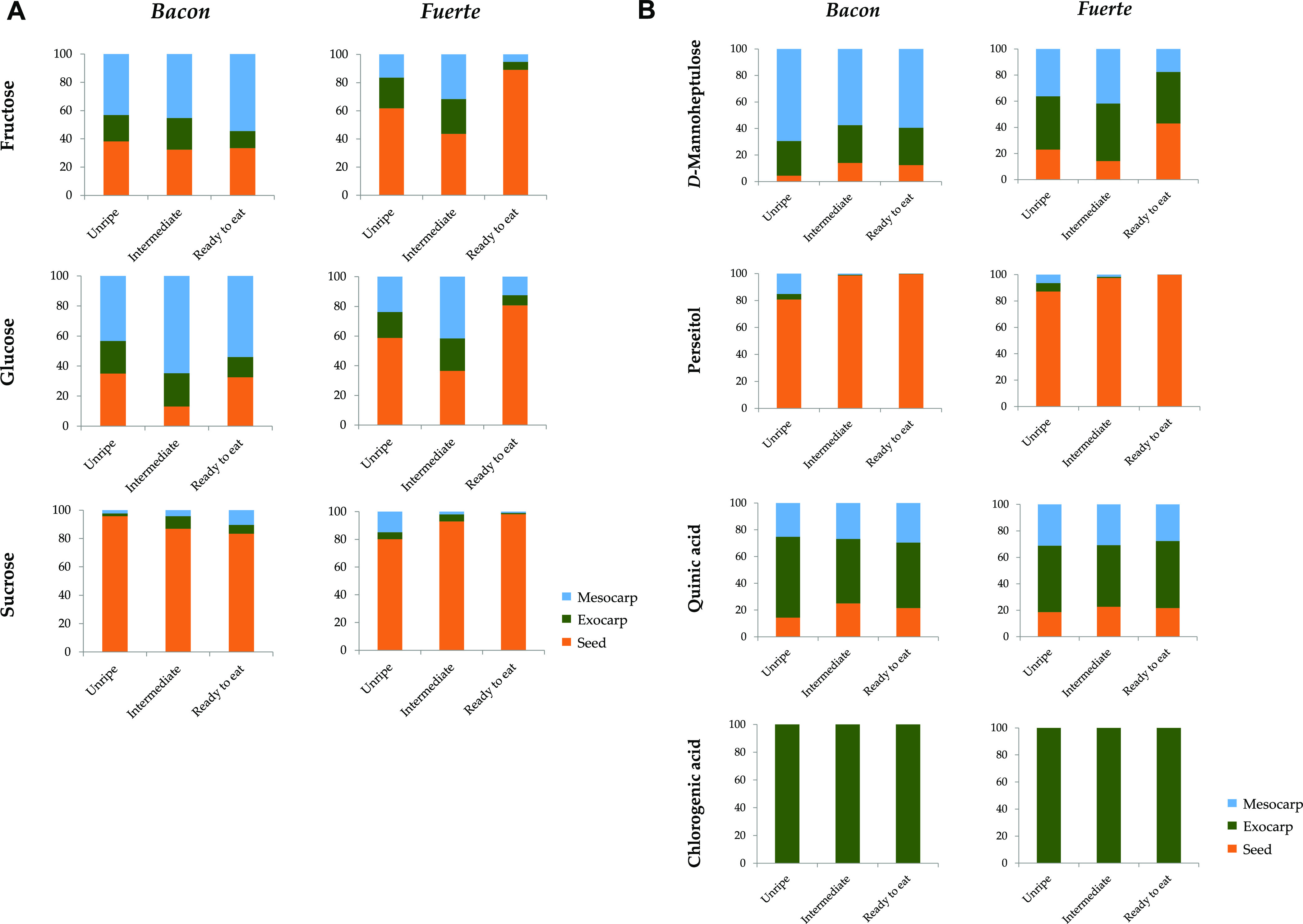
Distribution of each compound among the tissues studied at each ripening stage. Representations have been made for each metabolite showing the percentage of the total content of the fruit found in each tissue.

In order to establish the distribution of each compound among the tissues studied at each ripening stage, representations have been made for each metabolite, showing the percentage of the total content of the fruit found in each tissue. In Figure 2, the total sum of each compound was calculated considering the three tissues analyzed, and then, the percentage that the amount found in each matrix represented of the whole was estimated.

Among the three matrices, fructose was found mainly in mesocarp in *Bacon*, a fact that was more noticeable as the fruit ripened. In *Fuerte*, however, fructose was found in a greater proportion in the stone; this fact is more readily discernible in ripe avocados. Glucose (which is an isomer of fructose) presented a similar distribution to that of fructose for *Bacon* fruits, being predominant in the mesocarp. Among the different ripening stages, the percentage of glucose in mesocarp was higher in the intermediate phase (65%, completing the distribution with 13 and 22% in seed and exocarp, respectively). In *Fuerte*, glucose prevailed in the seed, particularly in ready-to-eat fruits. In both varieties, sucrose was found to be more abundant in seed tissues, with much lower concentrations in mesocarp and exocarp. This is in agreement with a previous study that determined, among other things, the sucrose concentration in various avocado tissues of the three varieties.^38^ However, Liu and co-workers reported very similar levels of sucrose in both mesocarp and seed of *Hass* avocado fruits.^13^

The proportion of d-mannoheptulose in *Bacon* mesocarp was higher than the sum of percentages found in seed and exocarp at the three ripening stages. In *Fuerte*, d-mannoheptulose was better distributed among the three matrices, increasing in the seed and decreasing in the mesocarp with ripening. In a previous study, it was determined that d-mannoheptulose levels in the mesocarp of *Hass* fruits were higher than those found in exocarp and seed.^38^ This would be homologous to what was observed in this study for *Bacon* variety. However, the same authors described that in the case of *Pinkerton* and *Fuerte* avocados, the most notable concentrations of d-mannoheptulose were found in the exocarp. The latter is what has been evidenced in the present study for unripe and “medium ripening” *Fuerte* avocados; in the case of ripe fruits, the concentrations of d-mannoheptulose in exocarp and seed were very similar.

The graphs for perseitol were practically identical for the two varieties at the three ripening stages. This compound was markedly preponderant in the seed, especially as the fruits became more mature. As previously stated, perseitol may act as a storage sugar (energy source) and d-mannoheptulose as a transport sugar and, in some cases, as an energy supplier in the production of other compounds. The conversion between the two takes place through an aldose enzyme present in the Calvin cycle. This transformation between aldoses allows the supply of transport sugars in the fruit mesocarp.^10,13^ It has also been indicated that this transport of sugar in the fruit is part of the mechanism that inhibits fruit ripening on the tree. Furthermore, it is believed that the accumulation of perseitol could be closely related to an increase in the synthesis of new C7 sugars.^10^ The results obtained reinforce the hypothesis that perseitol is a storage carbohydrate, so this can probably explain why the concentration is high in the seed and notably lower in the rest of the tissues. Most authors in this field share the hypothesis that C7 sugars are “multifunctional sugars” and further research is needed to shed light on this topic and to elucidate the metabolism of heptose carbohydrates in avocado. A study with a larger number of samples and varieties could confirm the hypothesis that C7 sugars are widely used during ripening in several functions and should be observed to decrease as senescence approaches, making them potential biomarkers of ripening in this fruit.

For both varieties and regardless of the ripening stage, quinic acid showed a predominance in the exocarp, where its concentration represented approximately 50% of the total concentration of the three matrices. As pointed out above, chlorogenic acid was found in quantifiable quantities exclusively in the exocarp. Ramos-Aguilar and co-workers also found no detectable levels of this compound in mesocarp for samples of ripe fruits from Mexican *Creole*.^41^

The involvement of C6 and C7 sugars in avocado ripening is irrefutable, so they fluctuate as this process takes place (in some tissues, it will be more noticeable than in others). The data presented indicate that these fluctuations will depend on the variety studied, and what is established for *Bacon*, for example, does not necessarily resemble in its entirety what is established for *Fuerte* (neither for *Hass*, if we compare it with what is established in the published literature). Thus, the hypothesis we propose, and which should be confirmed by future research, is that the carbohydrates (C6 and C7) in the mesocarp of *Fuerte* tend to be consumed in metabolic processes, while the sugars in the flesh of *Bacon* do not follow the same trend. On the contrary, most of them decrease, except for glucose and fructose, which show a slight increase. It is plausible that, even if all fruits belong to the same species, fluctuations and relative levels of primary metabolites may induce to distinguish one variety from another.

In conclusion, in the present work, the first HILIC–MS method for the determination of C6 and C7 sugars in avocado, together with chlorogenic acid and quinic acid, has been developed and validated. The method was applied to the study of 54 samples of different tissues of *Bacon* and *Fuerte* avocados at different stages of ripening. d-Mannoheptulose was the main component in the mesocarp and exocarp, and perseitol was predominant in the seed, followed by sucrose and d-mannoheptulose. The inter-tissue distribution of sucrose, perseitol, quinic acid, and chlorogenic acid was very similar for *Bacon* and *Fuerte* varieties and was not influenced by the ripening state, since no significant differences were found during the statistical analysis (*p* ≥ 0.05). However, a different situation was observed for fructose, glucose, and d-mannoheptulose, whose partitioning differed greatly between varieties and also during fruit softening, showing a value of *p* ≤ 0.05. Considering the important role played by sugars as biomarkers during the maturation and ripening processes, it would be interesting to carry out a more detailed investigation to complete the information provided in this contribution, including other varieties and perhaps more ripening stages that would allow to properly monitor the evolution over the avocado fruit ripening process.

This study has provided a reliable and simple to apply analytical approach for the simultaneous determination of carbohydrates and other compounds of interest in avocado. It has also described in detail the composition of different avocado tissues, the distribution of polar metabolites between tissues of the same avocado fruit, and whether this distribution is altered during ripening. All these aspects are essential to explore and better comprehend the physiology of this tropical fruit. In addition, the provision of valuable information on cultivars other than *Hass* is of great interest to diversify the production of avocado varieties in different regions of the world. Follow-up work on other varieties is necessary to have an ample knowledge of the diversity present in avocado germplasm.

## Supporting Information Available

Examples of interesting papers that have determined the metabolites considered within this study by applying different analytical methodologies; sample extraction conditions tested; different chromatographic conditions tested; chromatographic profile obtained by representing the extracted ion chromatograms of the compounds of interest; and the average composition of fructose, sucrose, glucose, d-mannoheptulose, perseitol, and quinic acid in avocado exocarp, seed, and mesocarp for *Bacon* and *Fuerte* varieties at each ripening level (PDF)
