# Supplementary material for: Characterization of the Polar Profile of Bacon and Fuerte Avocado Fruits by Hydrophilic Interaction Liquid Chromatography–Mass Spectrometry: Distribution of Non-structural Carbohydrates, Quinic Acid, and Chlorogenic Acid between Seed, Mesocarp, and Exocarp at Different Ripening Stages
Source: J Agric Food Chem. 2023 Mar 29;71(14):5674–85. doi: 10.1021/acs.jafc.2c08855 (PMC10103167; doi:10.1021/acs.jafc.2c08855)
Supplement: Supplementary file 1 — jf2c08855_si_001.pdf [file jf2c08855_si_001.pdf]

Supporting Information

Characterization of the polar profile of *Bacon* and *Fuerte* avocado fruits by HILIC-MS: distribution of non-structural carbohydrates, quinic acid and chlorogenic acid between seed, mesocarp and exocarp at different ripening stages

María Gemma Beiro-Valenzuela[1♦], Irene Serrano-García[1♦], Romina P. Monasterio[1,3], María Virginia Moreno-Tovar[1], Elena Hurtado-Fernández[2], José Jorge Gonzalez-Fernández[4], José Ignacio Hormaza[4], Romina Pedreschi[5,6], Lucía Olmo-García[1*], Alegría Carrasco-Pancorbo[1]

[♦]equally contributing authors

[1] Department of Analytical Chemistry, Faculty of Sciences, University of Granada, Ave. Fuentenueva s/n, 18071, Granada, Spain.
[2] Department of Biological and Health Sciences, Faculty of Health Sciences, Loyola University (Universidad Loyola Andalucía). Avda. de las Universidades s/n, 41704, Dos Hermanas, Sevilla.
[3] Instituto de Biología Agrícola de Mendoza (IBAM), UNCuyo - CONICET, Facultad de Ciencias Agrarias, Chacras de Coria, Mendoza, Argentina.
[4] Institute for Mediterranean and Subtropical Horticulture (IHSM La Mayora-UMA-CSIC), 29750, Algarrobo-Costa, Málaga, Spain.
[5] Pontificia Universidad Católica de Valparaíso, Facultad de Ciencias Agronómicas y de los Alimentos, Escuela de Agronomía, Calle San Francisco S/N, La Palma, Quillota 2260000, Chile.
[6] Millennium Institute Center for Genome Regulation (CRG), Santiago, Chile.

Table 1. Examples of interesting papers that have determined the metabolites considered within this study by applying different analytical methodologies. Papers have been ordered in alphabetical order (considering first author surname).

Matrix	Variety	Fruit ripening stage	Metabolites determined	Analytical method	Column	Ref.
leaves and fruit tissues (mesocarp, exocarp and seed)	*Hass*	unripe fruit November 2005 (early fruit set); March 2006-June 2006 (close to harvest date)	*D*-mannoheptulose and perseitol	HPLC-RID	Anion Exchange	I*
mesocarp	*Hass*	fast- and slow ripening; fruit ripened in 6 – 7 days and 12 – 13 days, respectively (post-harvest)	fructose, glucose, sucrose, *D*-mannoheptulose, and perseitol	HPLC-RID	ECM-Monosaccharide	32
mesocarp, exocarp and seed	*Pinkerton*, *Hass* and *Fuerte*	unripe fruit (May and June harvest)	*D*-mannoheptulose, sucrose and perseitol	HPLC-RID	Silica gel	38
mesocarp	*Hass*	unripe, medium-ripe and ready-to-eat	fructose, glucose, sucrose, *D*-mannoheptulose and perseitol	HPLC-ELSD	CP-Sil 88	40
mesocarp	*Pinkerton*, *Hass*, *Lamb Hass*, *Fuerte* and *Ryan*	ripe fruit (harvested in different growing seasons)	chlorogenic acid and *D*-mannoheptulose	HPLC-DAD GC-MS	UPLC - HSS C18	39
mesocarp	*Hass*	ready to eat	fructose, glucose, sucrose, *D*-mannoheptulose, and perseitol	HPLC-ELSD	Kromasil 100 5NH$_2$ column	9
mesocarp and exocarp	*Hass* and *Creole*	unripe	fructose, glucose, sucrose, *D*-mannoheptulose, perseitol and quinic and chlorogenic acid	HPLC-RID	Shodex SUGAR SC 1821 (for sugar) and Aminex HPX-87H ion-exchange column (for organic acids)	41
mesocarp	*Hass*	ready-to-eat	fructose, glucose, sucrose, *D*-mannoheptulose, perseitol and quinic and chlorogenic acid	HPLC-RID	Shodex SUGAR SC 1821 (for sugar) and Aminex HPX-87H ion-exchange column (for organic acids)	II*
exocarp	*Hass* and *Shepard*	unripe fruit	chlorogenic acid	HPLC-Q-TOF	Zorbax Eclipse Plus C18	III*
mesocarp	23 varieties (including *Bacon* and *Fuerte*)	ripe and unripe fruit	fructose, glucose, perseitol and *D*-mannoheptulose	LC-RID	Waters μ Bondapak/carbohydrate column	IV*
mesocarp	*Carmen* and *Hass*	during growth, development and maturation	*D*-mannoheptulose and perseitol	HPLC-RID	RCM–Monosaccharide	5
mesocarp, exocarp and seed	*Hass*	ready to eat	fructose, glucose, sucrose, *D*-mannoheptulose and perseitol	HPLC-RID	RCM–Monosaccharide	37
mesocarp and seed	*Hass*	unripe fruit	*D*-mannoheptulose and perseitol	HPLC-RID	RCM–Monosaccharide	10
mesocarp	*Hass*	unripe fruit	chlorogenic acid	HPLC-DAD	X-terra C18 reverse phase	V*
leaves, trunk cambium, stems, roots, flower parts and mature fruit (mesocarp, exocarp and seed)	*Hass*	unripe fruit	*D*-mannoheptulose and perseitol	HPLC-RID	Anion Exchange	13

Abbreviations used in the table in alphabetical order: DAD (diode-array detector); ELSD (evaporative light scattering detector); GC (gas chromatography); HPLC (high-performance liquid chromatography); LC (liquid chromatography); MS (mass spectrometry); Q (quadrupole); RID (refractive index detector); TOF (time of flight).

*These articles have not been cited in the main text of the manuscript, so they will be referenced below.

(I) Bertling, I.; Tesfay, S. Z.; Bower, J. P. Antioxidants in "Hass" Avocado. *South African Avocado Grow. Assoc. Yearb.* **2007**, *30*.

(II) Ramos-Aguilar, A. L.; Ornelas-Paz, J.; Tapia-Vargas, L. M.; Gardea-Béjar, A. A.; Yahia, E. M.; Ornelas-Paz, J. de J.; Ruiz-Cruz, S.; Rios-Velasco, C.; Ibarra-Junquera, V. Comparative Study on the Phytochemical and Nutrient Composition of Ripe Fruit of Hass and Hass Type Avocado Cultivars. *J. Food Compos. Anal.* **2021**, *97*, 103796. https://doi.org/10.1016/j.jfca.2020.103796.

(III) Salazar-López, N. J.; Domínguez-Avila, J. A.; Yahia, E. M.; Belmonte-Herrera, B. H.; Wall-Medrano, A.; Montalvo-González, E.; González-Aguilar, G. A. Avocado Fruit and By-Products as Potential Sources of Bioactive Compounds. *Food Res. Int.* **2020**, *138* (September), 109774. https://doi.org/10.1016/j.foodres.2020.109774.

(IV) Shaw, P. E.; Wilson, C. W.; Knight, R. J. High-Performance Liquid Chromatographic Analysis of D-Manno-Heptulose, Perseitol, Glucose, and Fructose in Avocado Cultivars. *J. Agric. Food Chem.* **1980**, *28* (2), 379–382. https://doi.org/10.1021/jf60228a040.

(V) Villa-Rodriguez, J. A.; Yahia, E. M.; González-León, A.; Ifie, I.; Robles-Zepeda, R. E.; Domínguez-Avila, J. A.; González-Aguilar, G. A. Ripening of 'Hass' Avocado Mesocarp Alters Its Phytochemical Profile and the in Vitro Cytotoxic Activity of Its Methanolic Extracts. *South African J. Bot.* **2020**, *128*, 1–8. https://doi.org/10.1016/j.sajb.2019.09.020.

Table 2-Supporting Information. Sample extraction conditions tested.

Extractant agent	Sample amount and extractant volume	Extraction system
➢ One extraction cycle with EtOH/H_2O 80:20 (v/v)<br>➢ One extraction cycle with EtOH/H_2O 60:40 (v/v)<br>➢ First extraction cycle with EtOH/H_2O 80:20 (v/v). Second extraction cycle with EtOH/H_2O 60:40 (v/v)<br>➢ Double extraction with EtOH/H_2O 60:40 (v/v)<br>➢ First extraction cycle with MilliQ water. Second extraction cycle with EtOH/H_2O 60:40 (v/v)<br>➢ Sample wash with hexane and double extraction with EtOH/H_2O 60:40 (v/v)	➢ 200-250 mg of tissue sample<br>➢ 6, 12 and 24 mL of extractant agent	➢ Ultrasonic bath for 30 min<br>➢ Vortex shaking for 30 min<br>➢ Bath 80 °C for 60 min

* The design of the protocols was made considering some of the already published studies on avocado.[6–11] All these articles have been duly cited in the references section of the main text of the manuscript.

Table 3-Supporting Information. Different chromatographic conditions tested.

Chromatographic Gradient♦	Flow (mL/min)	Injection volume (µL)	Columns
0 min: 5% A; 95%B - 21 min: 100%A; 0% B			
min: 0% A; 100% B - 20 min: 50% A; 50%B			Poroshell HILIC-120
0 min: 0% A; 100% B – 5 min isocratic elution - 15 min: 35% A; 65%B	0.2-0.4	2-10	Fortis HILIC-Diol
0 min: 10% A; 90%B – 5 min isocratic elution - 15 min: 35% A; 65%B			
0 min: 2% A; 98% B – 5 min isocratic elution - 15 min: 35% A; 65% B			

In all the cases, initial conditions were resumed and the column was re-equilibrated until constant pressure was reached.

*The design of the HILIC methodology was performed taking into account some of the already published studies on polar compounds with HILIC columns as well as the manufacturer's recommendations [12–14]. The first article has been duly cited in the references section of the main text of the manuscript. The other two are the following ones:

Tang, D. Q., Zou, L., Yin, X. X., & Ong, C. N. (2016). HILIC-MS for metabolomics: An attractive and complementary approach to RPLC-MS. Mass spectrometry reviews, 35, 574–600. https://doi.org/10.1002/mas.21445.

Virgiliou, C., Sampsonidis, I., Gika, H. G., Raikos, N., & Theodoridis, G. A. (2015). Development and validation of a HILIC-MS/MS multitargeted method for metabolomics applications. Electrophoresis, 36(18), 2215–2225. https://doi.org/10.1002/elps.201500208.

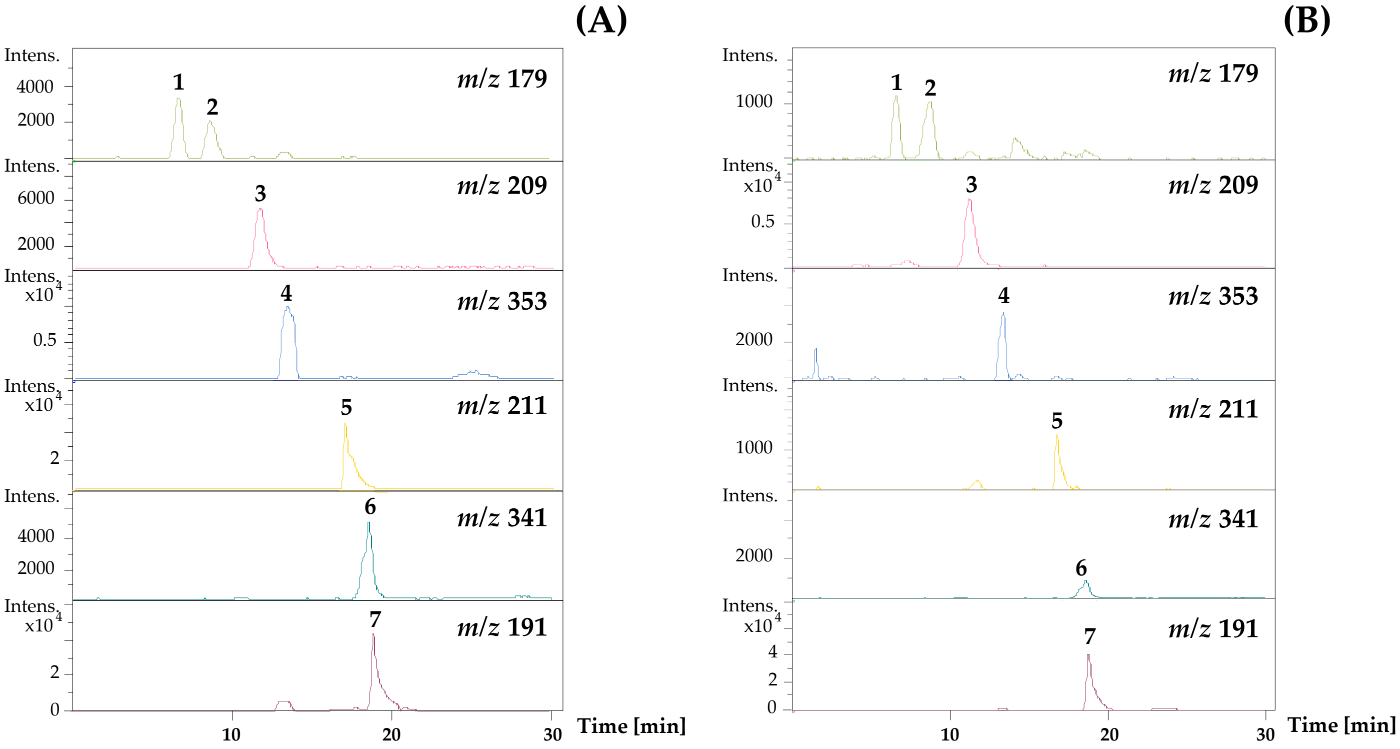

Figure 1-Supporting Information. Chromatographic profile obtained by representing the extracted ion chromatograms (EICs) of the compounds of interest. (A) mixture of pure standards and (B) example of peel avocado extract. Peaks identification: 1, fructose; 2, glucose; 3, *D*-mannoheptulose; 4, chlorogenic acid; 5, perseitol; 6, sucrose; and 7, quinic acid.

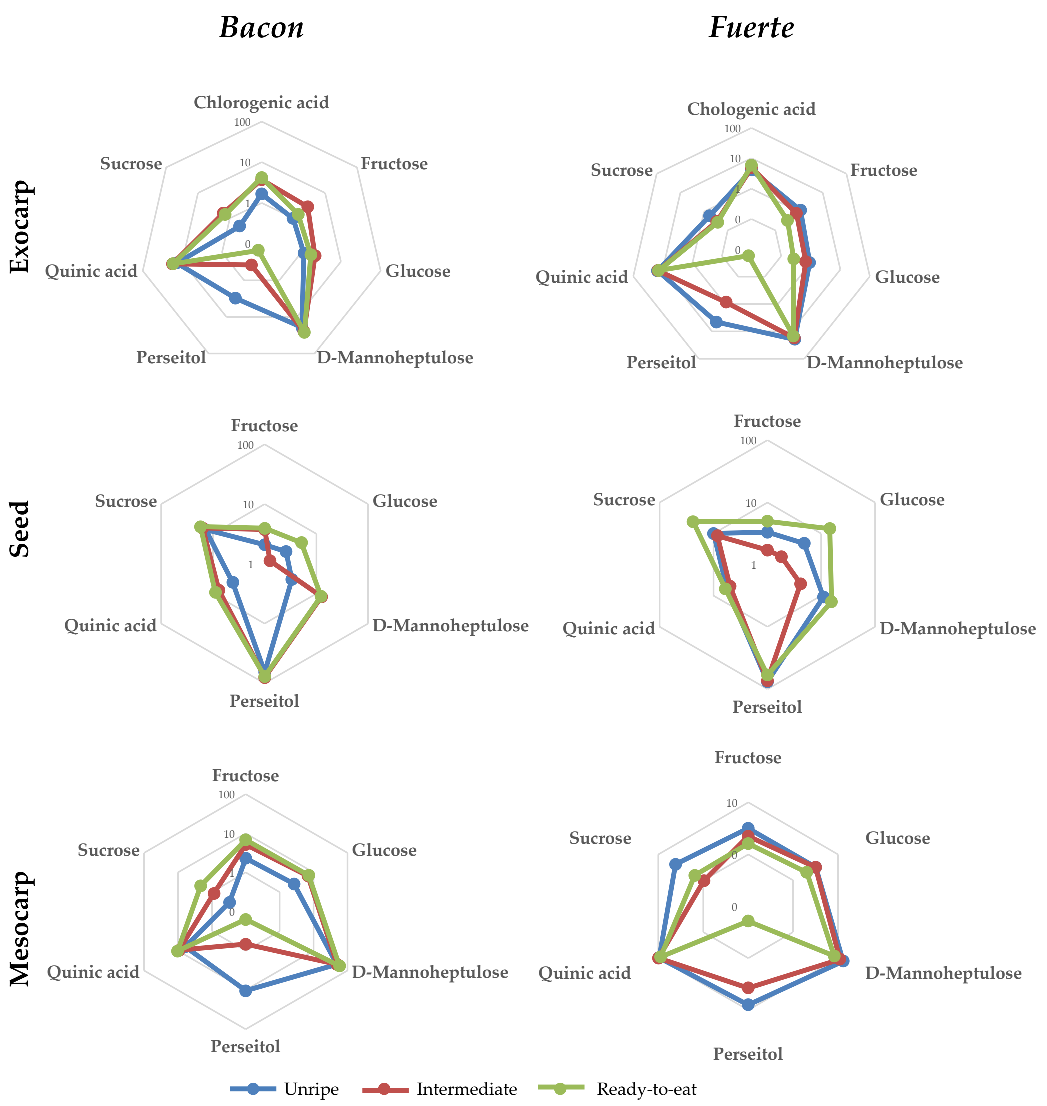

Figure 2-Supporting Information. Average composition of fructose, sucrose, glucose, *D*-mannoheptulose, perseitol and quinic acid in avocado exocarp, seed and mesocarp for *Bacon* and *Fuerte* varieties at each ripening level.
